# Influence of POSS Type on the Space Environment Durability of Epoxy-POSS Nanocomposites

**DOI:** 10.3390/nano12020257

**Published:** 2022-01-14

**Authors:** Avraham I. Bram, Irina Gouzman, Asaf Bolker, Nurit Atar, Noam Eliaz, Ronen Verker

**Affiliations:** 1 Department of Materials Science and Engineering, Tel-Aviv University, Ramat Aviv, Tel Aviv 6997801, Israel; neliaz@tauex.tau.ac.il; 2 Space Environment Department, Soreq Nuclear Research Center, Yavne 8180000, Israel; irina@soreq.gov.il (I.G.); asaf.bolker@gmail.com (A.B.); nurita.atar@gmail.com (N.A.); 3 Licensing & Safety Office, Israel Atomic Energy Commission, Tel Aviv P.O. Box 7061, Israel

**Keywords:** epoxy, polyhedral oligomeric silsesquioxane (POSS), low Earth orbit (LEO), outgassing, atomic oxygen (AO)

## Abstract

In order to use polymers at low Earth orbit (LEO) environment, they must be protected against atomic oxygen (AO) erosion. A promising protection strategy is to incorporate polyhedral oligomeric silsesquioxane (POSS) molecules into the polymer backbone. In this study, the space durability of epoxy-POSS (EPOSS) nanocomposites was investigated. Two types of POSS molecules were incorporated separately—amine-based and epoxy-based. The outgassing properties of the EPOSS, in terms of total mass loss, collected volatile condensable material, and water vapor regain were measured as a function of POSS type and content. The AO durability was studied using a ground-based AO simulation system. Surface compositions of EPOSS were studied using high-resolution scanning electron microscopy and X-ray photoelectron spectroscopy. It was found that with respect to the outgassing properties, only some of the EPOSS compositions were suitable for the ultrahigh vacuum space environment, and that the POSS type and content had a strong effect on their outgassing properties. Regardless of the POSS type being used, the AO durability improved significantly. This improvement is attributed to the formation of a self-passivated AO durable SiO_2_ layer, and demonstrates the potential use of EPOSS as a qualified nanocomposite for space applications.

## 1. Introduction

The low Earth orbit (LEO) space environment ranges from an altitude of 200 km up to an altitude of 1000 km [1]. It is characterized by extreme conditions such as hypervelocity micro-meteoroids, space debris, ionizing radiation, ultraviolet (UV) and vacuum UV (VUV) radiation, electrostatic discharge, extreme thermal cycles, atomic oxygen (AO), and ultrahigh vacuum environment (UHV) [2,3,4,5]. The pressure at LEO depends on the altitude and solar activity; at 160 km it is about 10^−6^ Torr while at 800 km it is about 10^−9^ Torr [6]. The UHV environment around the spacecraft leads to material outgassing (e.g., uncured molecules that diffuse from the bulk to the surface and outgas), which might result in dimensional change, mass loss, and contamination of sensitive surfaces [7,8,9,10]. The outgassed fragments that leave the surface can travel in the trajectory of the spacecraft and may collide with other outgassed molecules. As a result, they may backscatter and hit sensitive surfaces on the spacecraft. Thus, part of these fragments can stick and form a molecular layer that can contaminate and obscure optical devices [11]. Hence, any material intended to be used in space is required to successfully pass outgassing tests as per ASTM E595-15 [12] or ECSS-Q-ST-70-02C [13] standards.

Shape memory polymers (SMPs) are stimuli-responsive materials that, after deformation, can return to their pre-deformed shape by applying external stimuli [14,15,16,17,18]. SMPs have many advantages compared to common materials for space applications due to their excellent properties [19,20,21,22]. One of the most prominent groups of SMPs is based on epoxy resins [23,24,25,26,27]. Epoxy resins are high-performance thermoset polymers that exhibit outstanding mechanical properties (e.g., high modulus of elasticity and high creep resistance), high adhesion strength, good heat resistance, electrical insulation properties, and excellent resistance to chemicals [28,29]. Therefore, epoxy resins are one of the most common matrices in polymer-matrix composites (PMCs) for satellite applications [30,31]. In addition, SMP-based epoxy resins have the potential to replace metallic mechanisms in deployable systems for LEO spacecraft and can potentially be used in small satellites [17,18,32,33].

The most destructive element for organic materials being used in LEO space application is AO [34,35,36]. AO attack of organic materials can result in physical impinging and/or surface chemical reactions [31,37]. The principal reaction that the epoxy undergo with AO is assumed to be through the biphenyl segment containing a saturated alkyl bridge between the phenyl rings of the epoxy, and the alkyl substituents [31]. These reactions can lead to surface erosion that results in mass loss, changes in surface morphology and chemical composition, degradation of optical characteristics, as well as changes in the thermo-optical properties [38]. The AO fluence depends on the solar activity, orbit inclination, and the position of the material on the spacecraft vs. the spacecraft velocity vector [4]. The range of the AO density in LEO is 10^4^–10^9^ O-atoms/cm^3^ [1] and the AO flux ranges between 10^13^–10^15^ O-atoms/(cm^2^·s) [39]. Unprotected polymers will erode at a rate of ~100 µm per year due to hyperthermal AO impinging on their surface [40]. However, AO erosion affects only the top surface layer and, therefore, has minimum or no impact on the shape memory effect (SME) of SMPs exposed to the LEO environment [41].

Due to these extreme LEO environments, and especially due to the potential change in the thermo-optical properties of LEO-exposed materials, SMP-based epoxy resins are required to be protected against AO. Usually, this protection is provided to the spacecraft external materials by application of ~100 nm thick silicon dioxide (SiO_2_) or indium tin oxide (ITO) coatings. However, these protective coatings can be damaged due to hypervelocity debris, as well as by on-ground handling during the spacecraft integration processes [37]. A potential solution is to incorporate silicon into the polymer backbone [42]. Polyhedral oligomeric silsesquioxane (POSS) is a cage-like silicon oxide-based molecule that contains various organic functional groups [43]. Incorporation of POSS molecules with specific functional groups that react physically or chemically with a specific monomer can lead to a hybrid polymer-POSS material that has improved mechanical properties and lower AO erosion yield [44,45,46,47]. The low AO erosion yield in epoxy polymers containing POSS molecules is caused by AO oxidation of SiO_1.5_ POSS that leads to the formation of a passivated layer of SiO_2_ [30,48,49,50].

Recently, the effects of two types of POSS molecules on the shape memory and thermo-mechanical properties of epoxy-POSS (EPOSS) SMPs were investigated [51]. The first type, denoted as AM-EPOSS, contained different concentrations of POSS with amine (AM) functional groups. The second type, denoted as EP-EPOSS, contained different concentrations of epoxide (EP) functional groups.

In the present work, we studied the effect of incorporation of these two types of POSS molecules on the LEO space environment durability, using ground-based outgassing and AO exposure simulation facilities. To validate and comprehend the morphological and chemical mechanisms of the interaction between the EPOSS and AO, the surfaces of the EPOSS samples were characterized using high-resolution scanning electron microscopy (HRSEM) and X-ray photoelectron spectroscopy (XPS). A model that connects the EPOSS structure to its space durability properties is suggested.

## 2. Materials and Methods

### 2.1. Materials

**Figure 1:**
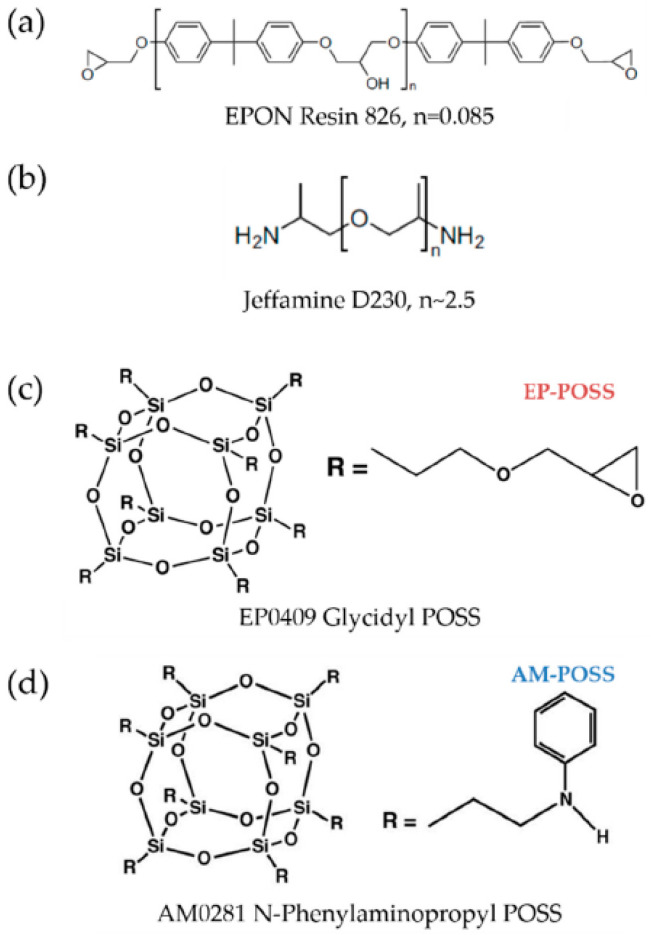
Molecular structure of (**a**) EPON 826 epoxy resin monomer, (**b**) Jeffamine D230 crosslinker, (**c**) EP0409 Glycidyl POSS (EP-POSS), and (**d**) AM0281 N-Phenylaminopropyl POSS (AM-POSS) [52,53,54,55].

Bisphenol A diglycidyl ether (DGEBA) epoxy monomer (EPON 826, from Hexion (Columbus, OH, USA) [52]), having an epoxide equivalent weight (EEW) of 182 g/eq, was used as the basic epoxy resin. Poly (propylene glycol) bis (2-aminopropyl) ether (Jeffamine D230, from Huntsman (The Woodlands, TX, USA) [53]), having an amine hydrogen equivalent weight (AHEW) of 60 g/eq, was used as the basic crosslinker. In addition, the following POSS reactants were used: N-Phenylaminopropyl POSS^®^ (AM0281, from Hybrid Plastics (Hattiesburg, MS, USA) [54]), with an AHEW of 186 g/eq, hereafter denoted as AM-POSS, or Glycidyl POSS^®^ (EP0409, also from Hybrid Plastics [55]), with an EEW of 167 g/eq, hereafter denoted as EP-POSS. The molecular structures of the EPOSS components are presented in Figure 1.

### 2.2. Preparation of Epoxy and EPOSS Samples

AM-POSS or EP-POSS reactants were used to substitute the Jeffamine D230 crosslinker or the epoxy EPON 826 resin, respectively. In both cases, a 1:1 molar ratio between the amine and the epoxide functional groups was maintained. The maximum concentration of POSS was reached by completely replacing the epoxy resin with EP-POSS, or by replacing the Jeffamine D230 crosslinker with AM-POSS. The various compositions of the EPOSS nanocomposites are given in Table 1. The difference between the maximum content of the AM-POSS and EP-POSS is caused by the difference between the amine and epoxide equivalent weight of Jeffamine D230 [53] and EPON 826 resin [52].

The samples were prepared in the following manner: The epoxy resin, crosslinker, and POSS reactants were weighed and transferred into a glass vial. First, the vial was heated to 80 °C and shaken by a vortex shaker for 1 min at 3000 rpm, thus producing a precured adhesive. Second, the adhesive was degassed for 8 min at a pressure lower than 10 Torr and a temperature of 80 °C. Third, the precured adhesive was casted into a disk-shaped aluminum mold pretreated with a Watershield™ (Zyvax^®^, Ellijay, GA, USA) release agent. The cavity dimensions were 12 mm diameter and 1 mm depth. Finally, the adhesive was cured at 100 °C for 1.5 h and then post-cured at 130 °C for 3 h [51].

### 2.3. Outgassing System

The outgassing properties of various EPOSS samples under vacuum and heat were determined according to ASTM E595-15 [12] and ECSS-Q-ST-70-02C [13] standards. According to these standards, a sample is first kept for 24 h of preconditioning in a humidity chamber maintained at 50% relative humidity (RH) at room temperature. Then, the sample is outgassed at a temperature of 125 °C while the pressure is kept lower than 5 × 10^−5^ Torr for 24 h. The sample is weighed before and after the test, and the outgassed (volatile) content from the sample is evaluated by the wt.% of the total mass loss (TML), before and after the experiment, out of the original sample mass. The sample mass was measured by a SE2 micro-balance (Sartorius, Göttingen, Germany) with a readability of 0.1 µg.

During the outgassing experiments, a collecting plate was kept at 25 °C to measure the wt.% of the collected volatile condensable material (CVCM) out of the original sample mass. After the outgassing process, the samples were transferred back to the humidity chamber for another 24 h. The volatile content, equivalent to water mass, was calculated by measuring the wt.% of water vapor regain (WVR) mass out of the original sample mass. The TML of the sample excluding the WVR was designated as the recovered mass loss (RML, wt.%). According to the ASTM E595-15 standard, the screening criteria require a TML of less than 1 wt.% and CVCM lower than 0.1 wt.% in order for a material to be considered for space applications. In comparison, according to the ECSS-Q-ST-70-02C standard, the screening criteria requires RML of less than 1 wt.% and CVCM lower than 0.1 wt.%.

### 2.4. AO Irradiation Facility

The durability of the samples to AO erosion was studied in accordance with the ASTM E2089-00 standard [56]. Initially, the samples were outgassed at room temperature and at a pressure lower than 10^−3^ Torr for 72 h. Following the outgassing stage, the samples were exposed to an environment simulating AO in a ground simulation system based on a LITMAS^®^ RPS, LB-3001 RF-oxygen plasma source (Advanced Energy, Boston, MA, USA). During the exposure experiments, each sample was placed on a holder inside the vacuum chamber, down-stream from the plasma source. The pressure inside the chamber was maintained at 2 × 10^−2^ Torr. The uniformity of the AO plasma source was confirmed by placing a Kapton sample near each EP-POSS sample. The mass loss of the Kapton samples was used to determine the AO equivalent fluence and flux. The equivalent AO flux that the RF plasma produced was at the order of 10^14^–10^15^ O-atoms/(cm^2^·s). For mass loss measurements, the samples were removed periodically from the system. Weight measurements were carried out using the Sartorius SE2 micro-balance. The AO equivalent fluence has been determined using the Kapton erosion yield (3 × 10^−24^ cm^3^/O-atom [57]), density (1.42 g/cm^3^ [58]), and its mass loss, resulted from AO exposure, see Equation (1) [56]. Then, after the AO equivalent fluence has been determined, the erosion yield of the EPOSS samples were calculated also according to Equation (1):

(1) $$F=\frac{\Delta m}{A\rho E}$$

where *F* is the AO equivalent fluence (O-atoms/cm^2^), Δ*m* is the mass loss (g), *A* is the sample’s exposed area (cm^2^), *ρ* is the material density (g/cm^3^), and *E* is the erosion yield (cm^3^/O-atom). The erosion yield was calculated from the mass loss of the sample after exposure to the total AO fluence.

### 2.5. Characterization Techniuqes

The change in the samples’ surface morphology as a function of AO flux was characterized using a Sigma 300 VP HRSEM from ZEISS (Oberkochen, Germany). XPS measurements were performed using a PHI 5600 Multi-Technique System (Physical Electronics, Chanhassen, MN, USA). The samples were irradiated with an Al K_α_ monochromatic source (1486.6 eV), and the emitted photoelectrons were analyzed by a spherical analyzer with a slit aperture of 0.8 mm. The samples were characterized by depth profiling using 5 kV argon ions at a sputter rate of 47.6 Å/min, as measured on a SiO_2_/Si reference sample. Sample charging was compensated with a charge neutralizer, and the binding energies were calibrated according to the C 1s reference line at 285 eV. High-resolution XPS spectra were taken at pass energy of 11.75 eV. Spectra analysis of the different XPS lines was carried out using CasaXPS software (Version 2.3.19PR.0, Casa Software Ltd., Teignmouth, UK) with a Gaussian–Lorentzian product function and a non-linear Shirley background subtraction [59]. The Gaussian–Lorentzian mixing ratio was taken as 0.3 for all lines.

## 3. Results and Discussion

### 3.1. Outgassing Properties

**Figure 2:**
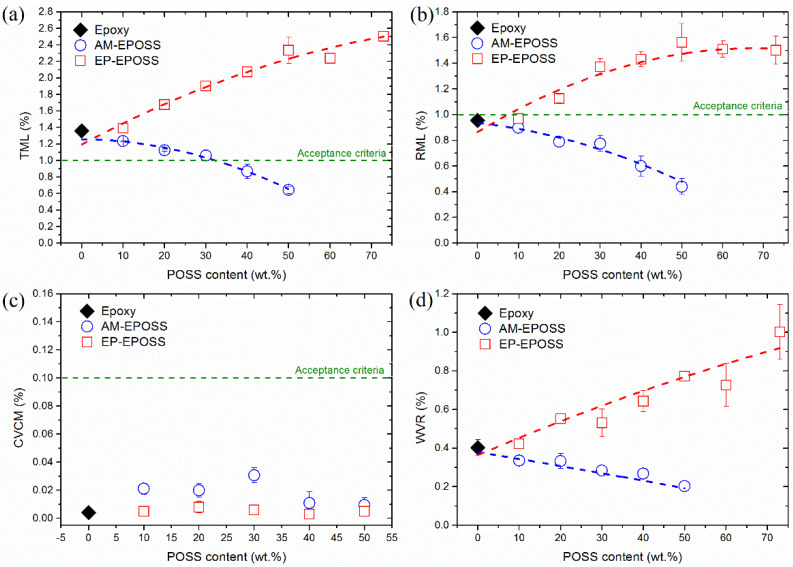
Effect of POSS type and content on: (**a**) TML, (**b**) RML, (**c**) CVCM, and (**d**) WVR values of EPOSS samples.

Figure 2 shows the effect of POSS type and content on (a) TML, (b) RML, (c) CVCM, and (d) WVR values of the EPOSS samples. It is evident that the pristine epoxy is not compatible with the ASTM E595-15 acceptance criteria due to a TML value larger than 1 wt.%, see Figure 2a [12]. However, it is compatible with the outgassing criteria of the ECSS-Q-ST-70-02C acceptance criteria, as its RML and CVCM are lower than 1% and 0.1%, respectively, see Figure 2b,c [13].

In the case of EP-EPOSS, as the EP-POSS content is increased, the TML, RML, and WVR values increase as well. On the other hand, increasing the EP-POSS content doesn’t affect the CVCM values, which are maintained at ~0.005 wt.%. The 10EP sample is the only EP-EPOSS composition that complies with the ECSS-Q-ST-70-02C acceptance criteria, albeit not with the ASTM E595-15 standard.

The increase in the TML and RML values, as the EP-POSS content was increased, may be associated with the lower degree of curing of the EP-EPOSS samples compared to pristine epoxy [51]. Under the vacuum and heat environment of the outgassing test, the lower degree of curing of the EP-EPOSS samples causes the release of molecular fragments that are not fully polymerized. This process is promoted by the increased presence of ether linkages in the EP-EPOSS polymer, which leads to an increase of the chain mobility and as a result, reduction in the *T*_g_ values [51,60,61]. As a result, uncured molecules can diffuse more easily from the bulk to the surface and outgas, resulting in higher TML and RML values [62]. The low CVCM results of all EP-EPOSS samples (Figure 2c) suggest that these outgassed fragments are volatile, but not condensable. Therefore, the molecular fragments that desorb from the EP-EPOSS surface are less likely to affect and cause degradation of optical surfaces, which may be located nearby on a spacecraft. The increase of the chain mobility as the EP-POSS content was increased can also explain the increase in the WVR values. As the chain mobility increases, water molecules can diffuse into the bulk more easily, increasing the WVR values.

As shown in Figure 2, all AM-EPOSS sample compositions are compatible with the outgassing acceptance criteria of the ECSS-Q-ST-70-02C standard. Moreover, AM-EPOSS samples with AM-POSS content above 30 wt.% also satisfy the ASTM-E595-15 outgassing acceptance criteria. Therefore, if these materials are used in the future on spacecraft, the AM-EPOSS compositions are less likely to affect and cause degradation of adjacent optical surfaces.

In the case of the AM-EPOSS samples, the trend is opposite to that of the EP-EPOSS samples—as the AM-POSS content was increased, the TML, RML, and WVR values decreased, see Figure 2a,b,d. On the other hand, similar to the case of EP-POSS, increasing the AM-POSS content did not affect the CVCM values, which remained at ~0.02 wt.% (see Figure 2c). According to previous work, as the AM-POSS content was increased, the AM-EPOSS degree of curing decreased sharply, resulting in shorter and less chemically crosslinked AM-EPOSS molecular chains [51]. This should have resulted in an increase in the TML and RML values. However, as the AM-POSS content was increased, *T*_g_ values were similar to the *T*_g_ of pristine epoxy for most of the compositions, and even slightly higher in the case of 50AM [51]. The reason for this is the increased presence of phenyl rings which increase the backbone rigidity of the network, increase the physical crosslinking density and *T*_g_, and decrease the chain mobility [63,64]. The lower chain mobility prevents uncured fragments from diffusing to the surface and outgassing from it [62]. It is assumed that when the AM-POSS content was increased, only uncured or partially cured molecules that were close to the surface were able to diffuse to it and to outgas, thus resulting in lower TML and RML values. In addition, according to the slightly higher CVCM values for AM-EPOSS compared to EP-EPOSS (see Figure 2c), the outgassing fragments from AM-EPOSS are more prone to condensate and are probably heavier than those from EP-EPOSS.

### 3.2. AO Durability

**Figure 3:**
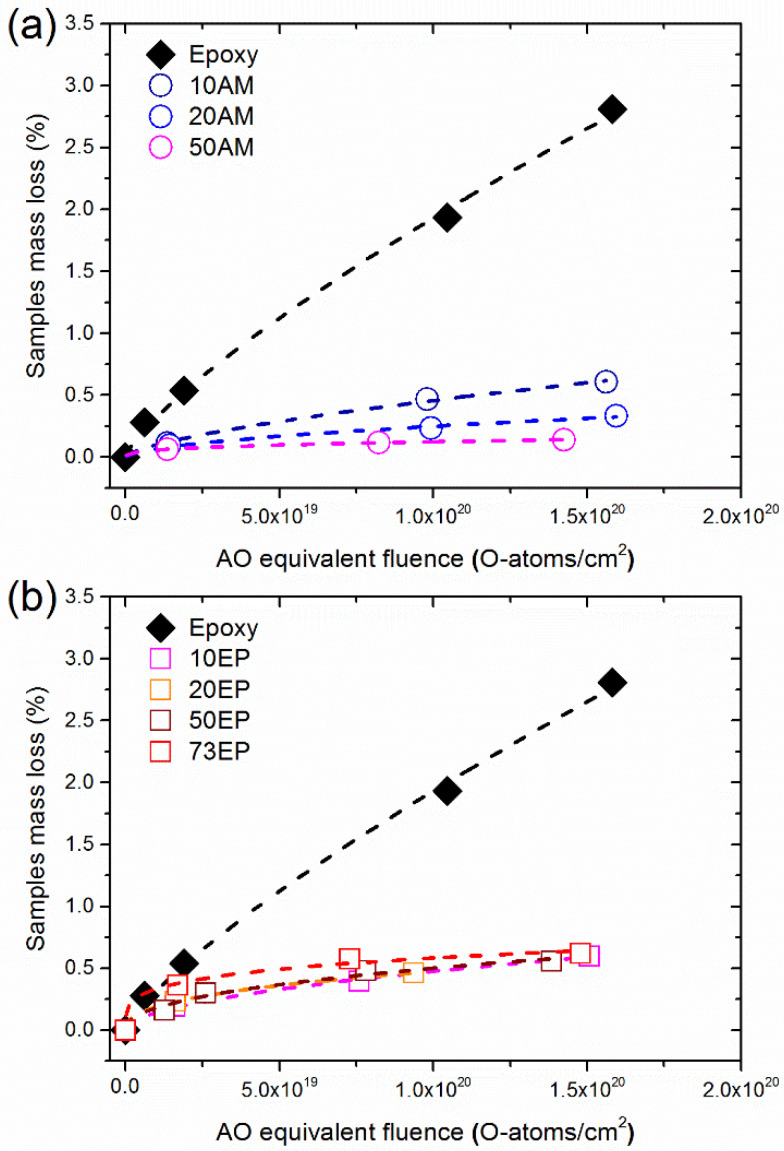
The mass loss of (**a**) AM-EPOSS, and (**b**) EP-EPOSS vs. AO equivalent fluence.

The mass loss (%) due to AO exposure of the AM-EPOSS and EP-EPOSS samples vs. AO equivalent fluence is shown in Figure 3. The mass loss of all EPOSS compositions decreased significantly compared to pristine epoxy. Thus, the AO durability of all POSS-containing samples improved substantially thanks to the addition of the POSS molecules. The mass loss rate of epoxy was linear, regardless of the AO fluence. The mass loss rates of the EPOSS samples were also linear above an AO equivalent fluence of 1 × 10^19^ O-atoms/cm^2^ but showed a much lower erosion rate pattern with the increase of the AO fluence. The lower erosion rate became more dominant as the amount of POSS was increased. This behavior indicates the formation of a SiO_2_ self-passivation layer on the EPOSS surfaces, which results in a decrease in the erosion rate [49].

**Figure 4:**
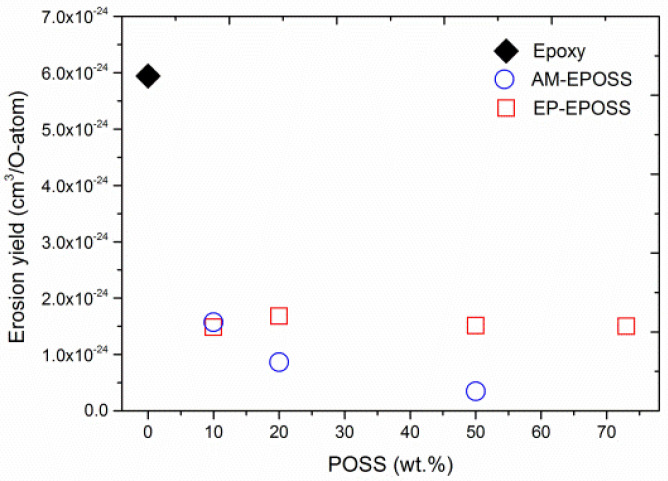
The erosion yield of epoxy, AM-EPOSS, and EP-EPOSS samples as a function of POSS content.

The erosion yield of the EPOSS samples is presented in Figure 4. The lower AO erosion yield of the samples containing POSS is thus attributed to the formation of a self-passivating layer of SiO_2_ [65]. It is evident that the erosion yield of AM-POSS samples decreased exponentially as the AM-POSS content was increased, from 6 × 10^−24^ cm^3^/O-atom in the case of epoxy, to 3 × 10^−25^ cm^3^/O-atom in the case of the 50AM sample. The addition of AM-POSS can therefore improve the epoxy durability to AO erosion by a factor of twenty. However, the erosion yield of the EP-EPOSS samples was only ~3 times smaller compared to the erosion yield of the epoxy and was less dependent on EP-POSS content. The reason for this difference lies in the different morphology of these two types of nanocomposites, as discussed below.

In general, ground simulation of the LEO hyperthermal AO environment by exposure to RF-plasma thermal AO has similar effects on organic materials [2]. However, previous ground-based erosion yield measurements of samples containing POSS molecules showed that such ground-based simulations are far more stringent than actual LEO conditions, showing 1–2 orders of magnitude higher erosion yield values compared to spaceflight experimental results [65,66,67]. These differences between ground-based simulation systems and spaceflight measurements become larger as the POSS content increases [65,67]. The self-passivation layer of the EPOSS samples is expected to improve significantly the effective AO durability of the samples. Therefore, the mass loss in LEO of the EPOSS samples is expected to be negligible even in the case of the EP-EPOSS samples.

**Figure 5:**
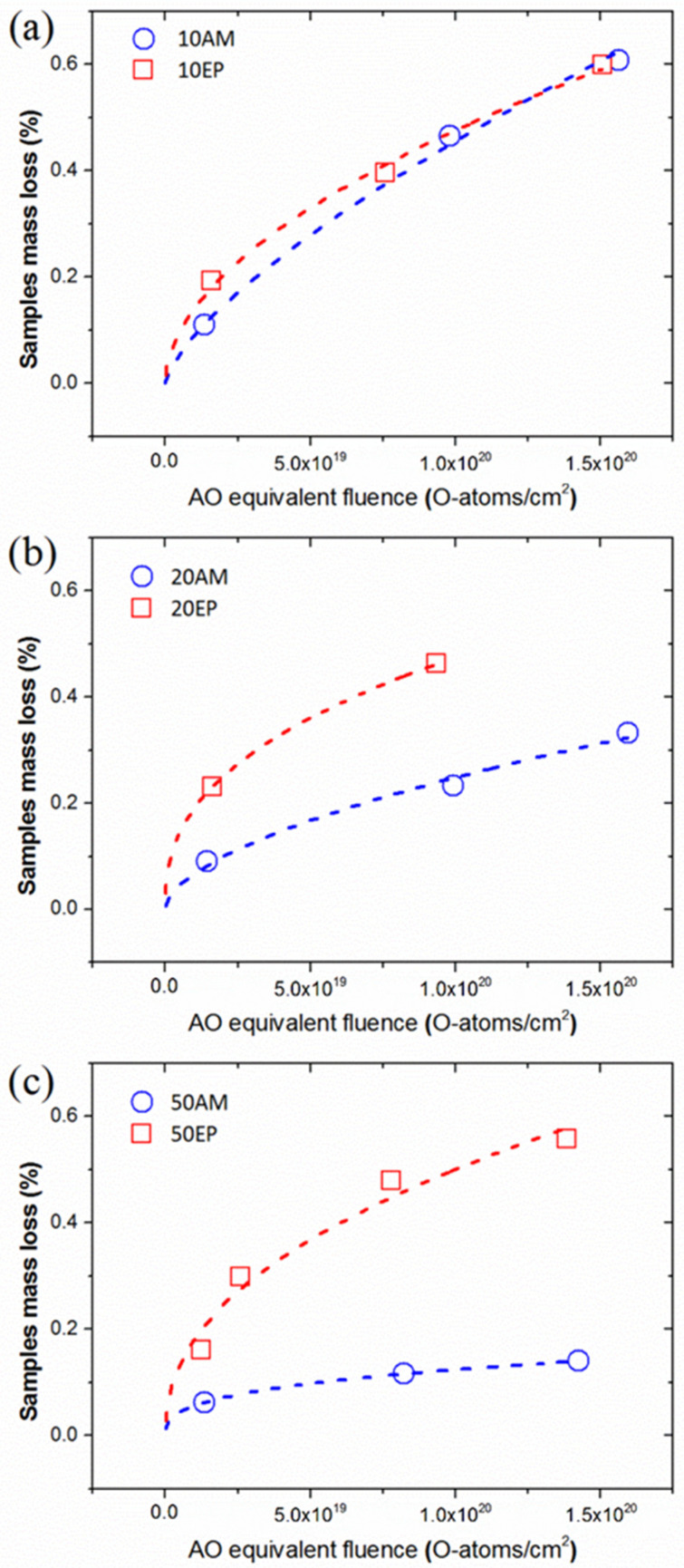
Comparison between the AM-EPOSS and EP-EPOSS mass loss versus AO equivalent fluence for: (**a**) 10 wt.%, (**b**) 20 wt.%, and (**c**) 50 wt.% POSS contents.

Figure 5 presents comparisons between the mass loss of AM-EPOSS and EP-EPOSS samples with the same amount of POSS vs. AO equivalent fluence. It is evident that the mass loss of 10 wt.% POSS was almost similar for both types of EPOSS samples (Figure 5a). However, with increasing POSS content to 20 and 50 wt.% (Figure 5b,c), the AM-EPOSS mass losses were two and three times smaller, respectively, compared to EP-EPOSS. In terms of AO durability, AM-EPOSS exhibited superior properties. These results are in line with the erosion yield values of the different compositions (see Figure 4). The erosion yields of EP-EPOSS samples were higher than AM-EPOSS samples. This difference can be attributed to the higher chain mobility in EP-EPOSS compared to AM-EPOSS. The higher chain mobility allows AO to diffuse more easily into the bulk of the material, causing accelerated erosion and a slower formation rate of the SiO_2_ passivation layer. Accordingly, the EP-EPOSS mass loss was higher than that of AM-EPOSS, until an effective passivation layer was formed on the surface. These results, combined with the outgassing tests results, lead to the conclusion that AM-EPOSS samples have superior properties compared to EP-EPOSS for space applications.

### 3.3. Surface Morphology and Chemical Composition

**Figure 6:**
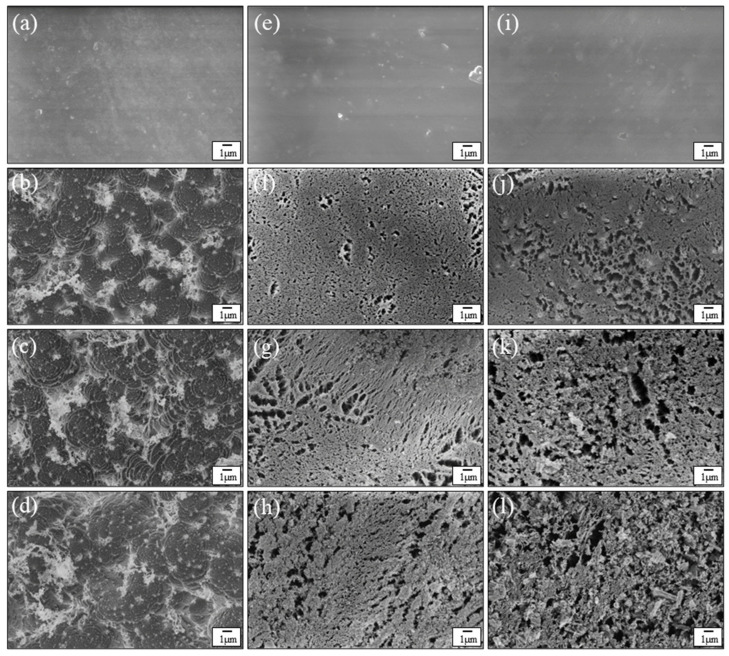
HRSEM images of (**a**–**d**) epoxy, (**e**–**h**) 20AM, and (**i**–**l**) 20EP samples before and after exposure to AO fluence. (**a**,**e**,**i**) are images of unexposed samples. (**b**,**f**,**j**) are images after AO equivalent fluence of 4.1 × 10^19^ O-atoms/cm^2^. (**c**,**g**,**k**) are images after AO equivalent fluence of 1.0 × 10^20^ O-atoms/cm^2^. (**d**,**h**,**l**) are images after AO equivalent fluence of 1.5 × 10^20^ O-atoms/cm^2^.

HRSEM images of the surface morphology as a function of AO equivalent fluence are depicted in Figure 6 for epoxy (a–d), 20AM (e–h), and 20EP (i–l) samples. Figure 6a,e,i shows the surface morphology of pristine samples. Figure 6b,f,j were taken after exposure of the samples to an AO equivalent fluence of 4.1 × 10^19^ O-atoms/cm^2^. Figure 6c,g,k were taken after exposure of the samples to an AO equivalent fluence of 1.0 × 10^20^ O-atoms/cm^2^. Figure 6d,h,l were acquired after exposure of the samples to an AO equivalent fluence of 1.5 × 10^20^ O-atoms/cm^2^.

The surface of the pristine samples is smooth and uniform, regardless of the POSS content (Figure 6a,e,i). In contrast, the surface morphology of the POSS-containing samples changed significantly after exposure to an AO equivalent fluence of 4.1 × 10^19^ O-atoms/cm^2^, in comparison to the surface morphology of epoxy (Figure 6b,f,j). At this fluence, the pristine epoxy surface was rough and formed a carpet-like morphology, which is typical of amorphous organic materials after exposure to AO [68]. From this point on, as the AO equivalent fluence was increased to 1.0 × 10^20^ and 1.5 × 10^20^ O-atoms/cm^2^, the surface morphology of the epoxy did not change significantly (see Figure 6c,d, respectively). These results are in agreement with the mass loss measurement results shown in Figure 3. This indicates that at these AO equivalent fluences, the epoxy erosion morphology was independent of the AO fluence.

The surface morphology of 20AM (Figure 6f–h) and 20EP (Figure 6j–l) samples after AO exposure (denoted as 20AM-AO and 20EP-AO, respectively) was sponge-like. However, the porosity of the 20EP was larger than that of 20AM. It seems that 20EP-AO eroded faster than 20AM-AO. This corresponds to the AO erosion kinetics measurements that were presented earlier, see Figure 5b. Moreover, as the AO equivalent fluence was increased, the cavities in the 20EP sample grew and their density was increased. This is attributed to the higher chain mobility in 20EP compared to 20AM, which results in higher AO diffusivity and increased AO erosion attack. The cavities in the 20EP are bigger and probably deeper than those in the 20AM sample. The inferior outgassing properties of EP-EPOSS compared AM-EPOSS (see Figure 2a) promoted the growth of cavities.

**Figure 7:**
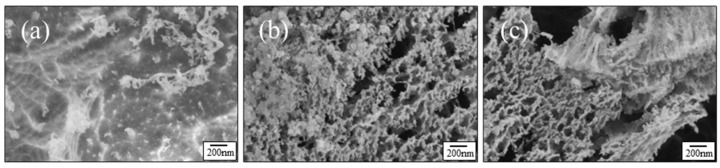
HRSEM images of the surfaces of (**a**) pristine epoxy, (**b**) 20AM, and (**c**) 20EP samples after exposure to an AO equivalent fluence of 1.5 × 10^20^ O-atoms/cm^2^.

High-resolution images of the surface morphology of the samples after exposure to AO equivalent fluence of 1.5 × 10^20^ O-atoms/cm^2^ are shown in Figure 7. These high-resolution images show more clearly the sponge-like morphology of the 20EP-AO and 20AM-AO samples.

**Figure 8:**
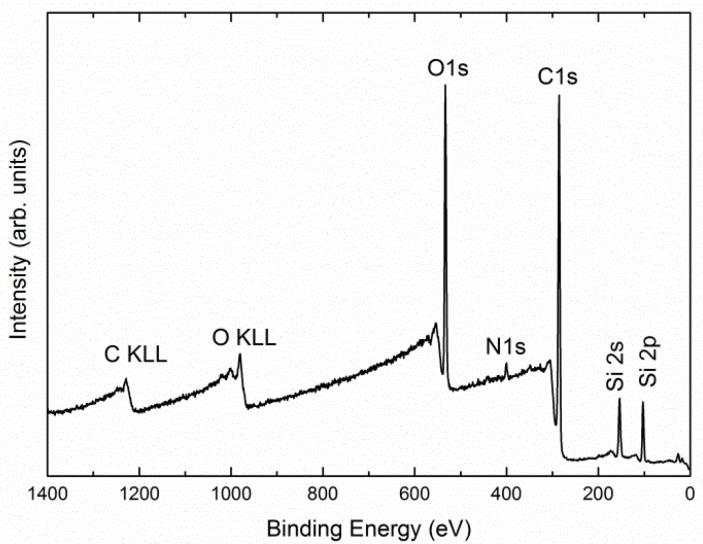
XPS survey spectrum of the pristine 20AM sample.

Figure 8 shows typical XPS survey spectrum of the 20AM sample. The spectrum reveals the core-level lines of carbon, oxygen, nitrogen and silicon, and the Auger lines of oxygen (O KLL) and carbon (C KLL). The same spectrum was obtained for the 20EP sample (not shown herein). The surface composition of the different samples was assessed by the analysis of C 1s, O 1s, and Si 2p core-level lines.

**Figure 9:**
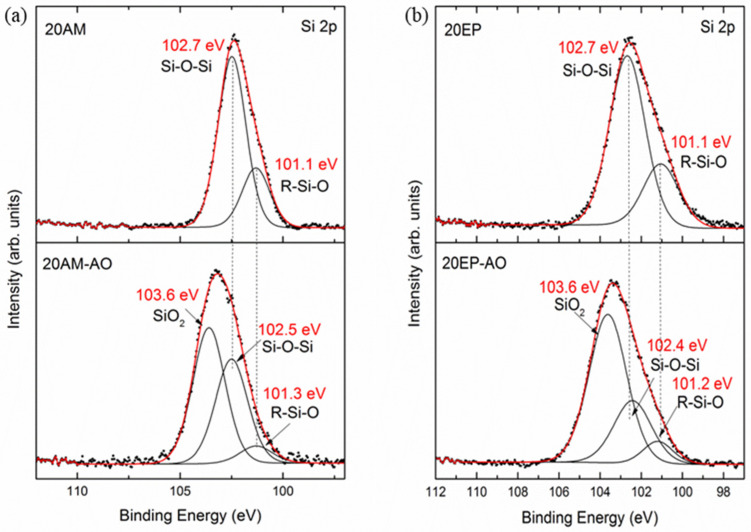
High-resolution XPS Si 2p core level lines measured for both pristine and AO exposed (**a**) 20AM and (**b**) 20EP samples.

Figure 9a,b show high-resolution XPS spectra of the Si 2p peak for 20AM and 20EP samples before and after RF oxygen plasma exposure. In the case of pristine 20AM and 20EM samples, the main Si 2p peak is positioned at a binding energy of 102.7 eV. After AO exposure, the Si 2p line shifts to a higher binding energy of 103.6 eV, which corresponds to the oxidation and formation of a SiO_2_ passivation layer on the sample surface [69]. XPS measurements of epoxy samples before and after exposure to the RF oxygen plasma (not shown herein) indicated the presence of Si-based surface contamination. Consequently, accurate curve-fitting and resolving the precise sample stoichiometry were complicated. The Si-O-Si and R-Si-O chemical bonds can be attributed to both the POSS chemistry and Si-based surface contamination. Nevertheless, this high-resolution XPS analysis clearly indicates that regardless of the nature of the POSS molecule added to the epoxy matrix—either AM-POSS or EP-POSS—exposure to AO resulted in the same surface chemistry, characterized by formation of a self-passivated SiO_2_ layer.

The oxidation of the POSS molecules and the formation of this passivation layer decreased the AO erosion rate of the POSS-containing samples. However, the presence of the Si-based contamination and its exposure to AO didn’t form a passivation layer, as indicated by the fast erosion rate of the epoxy (see Figure 3).

**Figure 10:**
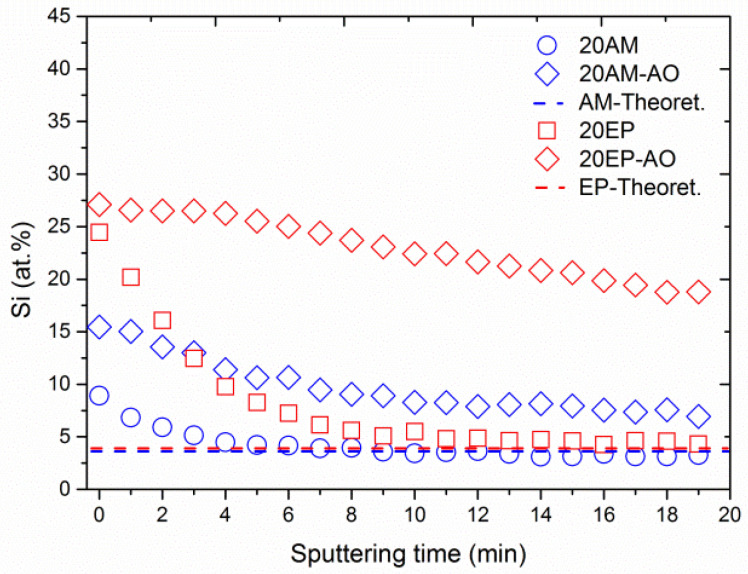
Depth profiling of the Si 2p peak for both pristine and AO-exposed 20AM and 20EP samples.

Figure 10 shows depth profiling of the Si 2p peak of both pristine and AO-exposed 20AM and 20EP samples vs. sputtering time. Figure 10 also presents the theoretical at.% Si for each of the two samples. In the case of the two pristine samples, Si-based surface contaminants, which had been adsorbed on the surface, were etched away within 10 min of the sputtering process, until a constant value was reached. The Si content of these pristine samples correspond to the theoretical Si content according to the POSS stoichiometry. In the case of EP-EPOSS, 4.3 at.% Si was measured, compared to the theoretical value of 3.9 at.%. In the case of the AM-EPOSS sample, 3.1 at.% Si was measured, compared to the theoretical value of 3.6 at.%. However, in the case of the AO-irradiated samples, 20EP-AO had almost double Si content than 20AM-AO at any given sputtering time. The higher chain mobility of EP-EPOSS enables AO to penetrate into the matrix more easily than in the case of AM-EPOSS. Taking into account that the outgassing properties of the EP-EPOSS samples promoted this trend, as apparent from the morphology of the AO-exposed samples (see Figure 6), it can be concluded that the porous, Si-rich, SiO_2_ passivation layer on the 20EP sample was thicker but less dense than that on the 20AM sample.

## 4. Conclusions

AM-EPOSS and EP-EPOSS samples were prepared with various POSS contents. The effects of the POSS type and content on the outgassing properties and AO durability of the various compositions were studied. It was found that for EP-EPOSS samples, as the POSS content was increased, the TML and RML outgassing properties deteriorated. However, the EP-POSS content did not have any influence on the CVCM value, which was found to satisfy the outgassing criteria. It is suggested that, as the EP-POSS content was increased the increased presence of ether linkages leads to an increase in the chain mobility. Thus, uncured molecules and small molecular fragments can diffuse more easily to the surface and outgas.

In contrast, in the case of the AM-EPOSS samples, the increase of AM-POSS content resulted in improved TML and RML outgassing properties, while the CVCM was almost constant and way below the outgassing acceptance criteria. The reason for this phenomenon is that as the AM-POSS content was increased the presence of phenyl rings increased too. This led to an increase in the backbone rigidity of the network and to a decrease of the chain mobility.

The lower chain mobility reduced the rate of diffusion of small molecular fragments towards the surface, thus decreasing the amount of outgassing species. In addition, according to the CVCM results, the outgassing fragments are more condensable, and probably heavier, in AM-EPOSS than in EP-EPOSS.

The EPOSS samples exhibited higher durability to AO erosion compared to pristine epoxy. The decreased erosion rate is attributed to the formation of a passivation layer due to AO oxidation of the SiO_1.5_ POSS into SiO_2_ layer. AM-EPOSS was found to have better AO durability compared to EP-EPOSS, and in that sense it is more suitable for LEO space applications. The EP-EPOSS exhibited lower AO durability, compared to AM-EPOSS, due to the higher chain mobility, which caused increased AO-induced damage deep below its surface. As a result, the SiO_2_ passivation layer on EP-EPOSS surface was thicker and more porous than that of AM-EPOSS.

The durability of AM-EPOSS to AO erosion improved with increasing AM-POSS content. However, in the case EP-EPOSS, no measurable change could be found in the erosion rate due to increase in EP-POSS content and exposure to AO for POSS contents of 10 wt.% and above.

This work presents novel SMP nanocomposite materials with excellent outgassing properties for most of the measured compositions. It demonstrates the ability to incorporate POSS molecules into the backbone of EPOSS to increase their AO durability. The diversity of the POSS types and contents and the associated material properties enable to promote the development of these materials for future SMP-based deployable space applications.

## Figures and Tables

**Table 1:** Composition of EPOSS nanocomposite samples.

Sample Name	wt.% EPON 826 Resin	wt.% D-230 Cross-Linker Agent	wt.% POSS
Epoxy	75	25	0
10AM	70	20	10
20AM	65	15	20
30AM	60	10	30
40AM	55	5	40
50AM	50	0	50
10EP	65	25	10
20EP	55	25	20
30EP	45	25	30
40EP	34	26	40
50EP	24	26	50
60EP	14	26	60
73EP	0	27	73
